# Incorporation of Superparamagnetic Iron Oxide Nanoparticles into Collagen Formulation for 3D Electrospun Scaffolds

**DOI:** 10.3390/nano12020181

**Published:** 2022-01-06

**Authors:** Manuel Estévez, Giorgia Montalbano, Alvaro Gallo-Cordova, Jesús G. Ovejero, Isabel Izquierdo-Barba, Blanca González, Clarissa Tomasina, Lorenzo Moroni, María Vallet-Regí, Chiara Vitale-Brovarone, Sonia Fiorilli

**Affiliations:** 1 Departamento de Química en Ciencias Farmacéuticas, Facultad de Farmacia, Universidad Complutense de Madrid, Instituto de Investigación Sanitaria Hospital 12 de Octubre i+12, 28040 Madrid, Spain; manestev@ucm.es (M.E.); blancaortiz@ucm.es (B.G.); vallet@ucm.es (M.V.-R.); 2 Department of Applied Science and Technology, Politecnico di Torino, 10129 Torino, Italy; giorgia.montalbano@polito.it (G.M.); chiara.vitalebrovarone@polito.it (C.V.-B.); 3 Department of Energy Environment and Health, Instituto de Ciencia de Materiales de Madrid C.S.I.C., Sor Juana Inés de la Cruz 3, Cantoblanco, 28049 Madrid, Spain; alvaro.gallo@csic.es (A.G.-C.); jgovejero@ucm.es (J.G.O.); 4 CIBER de Bioingeniería Biomateriales y Nanomedicina CIBER-BBN, 28040 Madrid, Spain; 5 Complex Tissue Regeneration Department, MERLN Institute for Technology-Inspired Regenerative Medicine, Maastricht University, Universiteitssingel 40, 6229 ET Maastricht, The Netherlands; c.tomasina@maastrichtuniversity.nl (C.T.); l.moroni@maastrichtuniversity.nl (L.M.)

**Keywords:** SPIONs, type-I collagen, magnetic scaffolds, electrospinning, human bone marrow-derived mesenchymal stem cells, bone regeneration, bone tissue engineering

## Abstract

Nowadays, there is an ever-increasing interest in the development of systems able to guide and influence cell activities for bone regeneration. In this context, we have explored for the first time the combination of type-I collagen and superparamagnetic iron oxide nanoparticles (SPIONs) to design magnetic and biocompatible electrospun scaffolds. For this purpose, SPIONs with a size of 12 nm were obtained by thermal decomposition and transferred to an aqueous medium via ligand exchange with dimercaptosuccinic acid (DMSA). The SPIONs were subsequently incorporated into type-I collagen solutions to prove the processability of the resulting hybrid formulation by means of electrospinning. The optimized method led to the fabrication of nanostructured scaffolds composed of randomly oriented collagen fibers ranging between 100 and 200 nm, where SPIONs resulted distributed and embedded into the collagen fibers. The SPIONs-containing electrospun structures proved to preserve the magnetic properties of the nanoparticles alone, making these matrices excellent candidates to explore the magnetic stimuli for biomedical applications. Furthermore, the biological assessment of these collagen scaffolds confirmed high viability, adhesion, and proliferation of both pre-osteoblastic MC3T3-E1 cells and human bone marrow-derived mesenchymal stem cells (hBM-MSCs).

## 1. Introduction

Bone disorders and injuries are considered a significant healthy and economic burden affecting millions of people worldwide and they are expected to rise due to the increasing aging of the population and the diffused sedentary lifestyle [1,2]. Despite the current pharmacological and surgical strategies that have demonstrated remarkable progress leading to the improvement of clinical outcomes, these approaches are limited by the poor availability of autologous tissues and the significant donor site morbidity [3,4]. Consequently, especially in pathological conditions, bone tissue engineering has gained great interest as an alternative method to support and stimulate the regeneration of new healthy tissue, where biomaterials and constructs are designed with the aim to guide and rebalance specific cell activities [5,6,7,8,9,10,11]. In this context, three dimensional (3D) scaffolds can be fabricated with the aim to mimic the chemical and morphological features of the native extracellular matrix (ECM), where different biomaterials can be selected and combined to provide specific and multiple stimuli to support the regeneration of the targeted tissue [5].

Among the different existing biomaterials, superparamagnetic iron oxide nanoparticles (SPIONs) have been widely explored in several biomedical applications such as magnetic resonance imaging, cell separation and detection, tissue repair, magnetic hyperthermia, drug and gene delivery [12,13,14,15]. Thanks to their superparamagnetic properties, size, biocompatibility, multifunctionality and possibility of further surface modification with various chemical agents, SPIONs represent a promising tool in many fields of medicine [16,17]. Furthermore, the absence of residual magnetization upon removal of an external magnetic stimulus allows for their precise remote control [16,17]. In this framework, SPIONs are particularly useful in regenerative medicine for labelling, tracking, and activation of stem cells [17], since their incorporation into the cells allows remote manipulation upon the application of an external magnetic field gradient. This facilitates the targeting of cells to desired sites for tissue regeneration [18,19], making SPIONS a powerful non-invasive tool in stem cell therapy [20].

With regard to cell activation, different physical forces such as fluid flow, axial compression, tension and magnetism can be used to generate a mechanical stimulus able to promote stem cell proliferation and differentiation [21,22,23]. These biological responses are achieved through the process of mechanotransduction, whereby cells convert mechanical stimuli into biochemical signals to generate a biological response [24,25]. In this scenario, the use of nanoparticles functionalized with antibodies or peptides targeting ion channels and membrane receptors such as integrins proved to elicit receptor activation and subsequent second messenger signals in human mesenchymal stem cells (hMSCs) [26]. Moreover, following the activation of SPIONs with an oscillating external magnetic field, the differentiation of osteoprogenitor cell populations towards an osteogenic lineage can be favoured [21,27]. In this regard, SPIONs have also demonstrated great potential in the design of magnetic scaffolds for different tissue engineering applications [28,29], especially in the regeneration of bone tissue [30,31], where improved implant integration and newly developed tissue with higher density have been achieved by applying an external magnetic field [32].

The advantages provided by the use of SPIONs can be further enhanced by exploiting their combination with polymeric matrices in order to design smart constructs able to support specific regeneration processes [33,34,35]. In this view, Johnson and co-workers observed the ability of poly-L-lactic acid (PLLA) electrospun fibrous scaffolds incorporating SPIONs to support neuron guidance, increasing neurite alignment and length within an injectable hydrogel [33].

For applications in bone tissue regeneration, type-I collagen represents a strategic candidate for the fabrication of biomimetic scaffolds since it represents the main organic phase of bone, in addition to the well-known high biocompatibility and ability to promote cell adhesion and proliferation [36,37,38]. However, the low mechanical properties and its high susceptibility to degradation and denaturation often require additional crosslinking treatments as well as the proper optimization of the manufacturing process to increase the stability of the final constructs [38,39,40].

Alongside the chemical composition to reproduce the structural features of native ECM, the electrospinning technique is considered a powerful tool to create biomimetic fibrous scaffolds. Electrospun scaffolds possess fibers ranging from the nano- to the micro-scale and a high surface-area-to-volume ratio, according to the physicochemical properties of the materials and the processing parameters [41,42].

Based on the above considerations, the present study aims at the fabrication of collagen-based electrospun scaffolds provided with magnetic properties through the incorporation of SPIONs into polymeric fibers during the electrospinning process. To the best of our knowledge, the combination of SPIONs with type-I collagen and the further processing of the resulting hybrid formulation by electrospinning technology have not been explored yet.

Firstly, in this work, uniform SPIONs were produced by a thermal decomposition process and transferred to the water by ligand exchange with dimercaptosuccinic acid (DMSA), obtaining time-stable colloidal suspensions of SPIONs. Subsequently, the developed SPIONs were combined with a type-I collagen solution and the processability of the hybrid formulation was explored by means of electrospinning. The obtained composite scaffolds were characterized and compared with SPIONs-free electrospun collagen mats. The morphological evaluation of the scaffolds was performed using scanning electron microscopy (SEM) and the distribution of the nanosized superparamagnetic particles throughout the collagenous matrix was observed by transmission electron microscopy (TEM), while vibrating sample magnetometry measurements were carried out to investigate the preservation of the magnetic properties. Finally, a preliminary biological assessment of the scaffolds was performed using pre-osteoblastic MC3T3-E1 and hBM-MSCs, to prove their biocompatibility and their ability to be cell colonized as potential platform for bone tissue regeneration.

## 2. Materials and Methods

### 2.1. Reagents

Sodium oleate 82%, FeCl_3_·6H_2_O 97%, oleic acid 90%, octadecene 90%, dimercaptosuccinic acid 98% (DMSA), dimethyl sulfoxide ≥99.9% (DMSO), acetic acid, and 12 kDa cellulose membrane were purchased from Sigma-Aldrich (Madrid, Spain). Absolute ethanol 99.5%, hexane 95% and toluene 99.5% were purchased from PanReac (Barcelona, Spain). All other chemicals (NaOH, HNO_3_ 65%, HCl 37%, NaCl, MgCl_2_, etc.) were of the highest quality commercially available and used as received. Milli-Q water (resistivity 18.2 MΩ·cm at 25 °C) was used in all experiments. Type-I collagen powders extracted from rat tails (N_COL) were provided by NOVAICOS Srl (Novara, Italy, https://www.novaicos.com/ (accessed on 20 December 2021)).

### 2.2. Preparation of SPIONs

Iron oxide nanoparticles were synthetized through thermal decomposition of an iron-containing precursor following a previously described procedure [43].

#### 2.2.1. Synthesis of Iron (III) Oleate

A total of 45 g of sodium oleate (82%) and 10.8 g of FeCl_3_·6H_2_O (97%) were added to a mixture of 60 mL of distilled water, 80 mL of absolute ethanol and 140 mL of hexane. The mixture was heated to 70 °C and vigorously stirred for 4 h. After cooling down to room temperature, the orange-brown organic phase was washed with ethanol/water mixtures: 1 × (15 mL:35 mL) plus 2 × (25 mL:25 mL). Hexane and traces of water and ethanol were removed in a rotary evaporator resulting in an iron (III) oleate as a dense product that was stored in an oven at 50 °C.

#### 2.2.2. Synthesis of Iron Oxide Nanoparticles

In the absence of oxygen, 4.5 g of iron (III) oleate were mixed with 0.71 g of oleic acid (90%) in 50 mL of octadecene (90%) in a 250 mL three-neck round bottom flask equipped with a reflux condenser, mechanical stirring (stirrer shaft), nitrogen flow and thermometer. The mixture was stirred at 340 rpm and heated to boiling temperature. When the temperature reached 50 °C, the stirring was stopped and the nitrogen flow was shut off at 100 °C. Once reflux was reached (315 °C), the reaction was kept at this temperature for 1 h and then allowed to cool to room temperature. The resulting product was washed with ethanol several times until a clean supernatant was obtained (ca. 15 × 30 mL using centrifugation cycles at 7500 rpm for 15 min). The material was dried under an air stream and resuspended in toluene.

#### 2.2.3. Transfer to Aqueous Medium by Ligand Exchange

A suspension of 50 mg of Fe_3_O_4_ nanoparticles in 20 mL toluene was added to a solution of 200 mg of DMSA (98%) in 5 mL of DMSO. The obtained suspension was placed in several glass vials that were shaken on a carousel (rotary shaker) at a constant speed for 3 days. DMSA-coated nanoparticles precipitated adhering to the wall of the glass vial, and the supernatant was discarded. The nanoparticles were then recovered and washed 4 times with ethanol by centrifugation at 7500 rpm for 20 min. The black solid was dried under an air stream and resuspended in 5 mL of distilled water. The pH of the suspension was adjusted to 10 with 2 M NaOH and dialyzed against distilled water for 72 h using a 12 kDa cellulose membrane. Finally, the pH of the colloidal suspension was adjusted to 7 and passed through a 0.22 µm filter.

### 2.3. SPIONs Characterization

The crystalline structure of the nanoparticles was analyzed by X-ray diffraction (XRD) with a Bruker D8 Advance diffractometer (Billerica, MA, USA) equipped with a graphite monochromator using CuKα radiation (λ = 1.5406 Å), within 10 and 90 2*θ* degrees. Particle size and shape were determined by transmission electron microscopy (TEM) using a JEOL JEM 1400 instrument operated at 120 kV (JEOL Ltd., Tokyo, Japan). Sample preparation was performed by placing one drop of a dilute suspension in toluene onto a carbon-coated copper grid. A vibrating sample magnetometer MagLabVSM (Oxford Instrument, High Wycombe, UK) was used to measure the magnetic properties of the nanoparticles before and after transfer to the aqueous medium. For the measurement, 25 µL of each sample were placed on a piece of cotton wool, allowed to dry at 50 °C and pressed into a sample holder. The hysteresis loops were measured at 290 K and 5 K up to 3 T, and the maximum magnetization at the maximum field was obtained for comparison. The hydrodynamic size was measured by dynamic light scattering (DLS) from a dilute suspension of the sample in water at pH 7. Electrophoretic mobility measurements for the nanoparticles suspended in water were used to calculate the zeta-potential (ζ) value of the DMSA-coated nanoparticles. Both DLS and electrophoretic mobility measurements were performed in a Zetasizer Nano ZS (Malvern Instruments Ltd., Worcestershire, UK) equipped with a 633 nm “red” laser. Thermogravimetric analysis (TGA) and differential thermal analysis (DTA) were performed in a Perkin Elmer Pyris Diamond TG/DTA analyzer (Perkin Elmer, CA, USA) by placing approximately 5 mg of sample in a platinum crucible and heating to 800 °C at 5 °C/min under an airflow rate of 100 mL/min. Fourier transformed infrared (FTIR) spectra were collected in a Thermo Nicolet Nexus spectrometer (Waltham, MA, USA) equipped with a Goldengate attenuated total reflectance (ATR) device. The Fe content in the samples was determined by inductively coupled plasma-optical emission spectrometry (ICP-OES) in a PerkinElmer Optima 2100 DV ICP apparatus (Waltham, MA, USA). Prior to the analysis, samples were digested at 90 °C in HNO_3_/HCl (1:3) overnight and diluted with double distilled water. The same procedure was followed to detect the iron content on the SPIONs containing collagen scaffolds.

### 2.4. Preparation of SPIONs Containing Collagen Formulation

To obtain the hybrid formulation, SPIONs were firstly homogeneously dispersed in 40% acetic acid in the water, sonicated for 1 h and stirred for 4 h. Subsequently, type-I collagen powders (N_COL) were dissolved into the particle suspension previously stirred overnight at room temperature, obtaining a final formulation containing a concentration of 20% (*w*/*v*) in collagen and a nominal 2% (*w*/*w*) of SPIONs with respect to the collagen weight (20% N_COL/2% SPIONs). In addition, a collagen solution (20 wt%) was prepared to dissolve rat tail collagen powders in 40% acetic acid (20% N_COL), as a reference for the electrospinning tests and the characterization of the designed scaffolds. The resulting 20% N_COL/2% SPIONs suspension and 20% N_COL solution were used for electrospinning tests immediately after preparation.

### 2.5. Rheology

The rheological assessment of the resulting hybrid formulation was carried out using a DHR-2 controlled stress rotational Rheometer (TA Instruments, Waters, New Castle, DE, USA). For the analysis, the instrument was equipped with a parallel plate geometry of 20 mm in diameter, while a Peltier plate system was exploited to constantly control the sample temperature. Flow ramp tests at 23 °C were conducted to investigate the viscosity of the 20% N_COL/2% SPIONs suspension over a wide range of shear rates (0.01–1000 s^−1^) and compared with that of the reference 20% N_COL solution.

### 2.6. Electrospinning of Col/SPIONs Scaffolds

Electrospun scaffolds of 20% N_COL/2% SPIONs and 20% N_COL (as reference) were obtained using a LE-50 Fluidnatek electrospinning system (Bioinicia, Spain) equipped with a temperature and humidity control system. A plate collector and a spinneret tip of 22 G (0.4 mm internal diameter) were used for all the tests to obtain randomly oriented fibrous mats. The electrospinning process was conducted setting a distance between the spinneret tip and collector of 12 cm (working distance) with voltage and flow rate ranging from 20 to 22 kV and 400 to 300 μL/h respectively, in order to obtain a stable material jet. The electrospinning was carried out for 3 h, keeping a constant temperature of 23 °C at 30% humidity in the working chamber. The resulting electrospun matrices were left to dry overnight at room temperature and subsequently chemically crosslinked with a solution of 30 mM 1-ethyl-3-(3-dimethylaminopropyl) carbodiimide-hydrochloride (EDC) and 15 mM N-hydroxysuccinimide (NHS) in ethanol, following a protocol previously optimized by Ribeiro et al. [44]. In brief, electrospun scaffolds were immersed in the EDC/NHS crosslinking solution at 4 °C for 8 h. The scaffolds were subsequently washed with ethanol to remove any crosslinker residuals and dried before analysis.

### 2.7. Characterisation of the Electrospun Scaffolds

#### 2.7.1. Morphological Assessment

Scanning electron microscopy (SEM) was used to explore the morphological features and measure the fiber diameter of the resulting 20% N_COL/2% SPIONs scaffolds. For the analysis, samples of the electrospun scaffolds were sputter-coated with platinum (up to 7 nm thickness), and images were acquired using a Desktop SEM Phenom XL (Phenom-World B.V., Eindhoven, The Netherlands) at an accelerating voltage of 15 kV and different magnifications.

The distribution of SPIONs into the collagen fibers of the scaffolds was observed by means of Transmission Electron Microscopy (TEM). For the analysis, the 20% N_COL/2% SPIONs scaffolds were embedded in Epon 112 for 72 h at 60 °C. Subsequently, ultrathin 50 nm sections were cut with a diamond knife on a Leica EM UC7 microtome and placed on copper TEM grids. Finally, samples were counterstained with 2% uranyl acetate for 30 s and imaged with a FEI Tecnai G2 Spirit BioTWIN iCorr microscope.

#### 2.7.2. Magnetic Properties

Vibrating sample magnetometry (VSM) was used to measure the magnetic properties of the collagen scaffold with the SPIONs incorporated. Circular samples were cut to have a diameter of 4 mm, accurately weighed and pressed into a sample holder. The hysteresis loops were measured at 290 K and 5 K up to 3 T, and the maximum magnetization at the maximum field was obtained for comparison.

### 2.8. In Vitro Cytocompatibility Tests

Phosphate buffered saline (PBS, pH 7.4), Dulbecco’s Modified Eagle’s Medium (DMEM), fetal bovine serum (FBS), penicillin-streptomycin, L-glutamine, trypsin/0.25% EDTA, Eagle’s minimum essential medium (α-MEM) and Triton X-100 were purchased from Gibco, Thermo Fisher Scientific, Wilmington, DE, USA. AlamarBlueTM HS Cell Viability Reagent, Atto 565-conjugated phalloidin and 4′-6-diamino-2′-phenylindole dihydrochloride (DAPI) were purchased from Invitrogen (Thermo Fisher Waltham, MA, USA). All other chemicals (paraformaldehyde, sucrose, hexamethyldisilazane, 4-(2hydroxyethyl)-1-piperazine-ethanesulphonic acid (HEPES), etc.) were of the highest quality commercially available and used as received.

For the in vitro tests, scaffolds were peeled off from the supporting aluminum foil and cut in pieces of about 20 × 20 mm^2^ to enable the punching of disks of 18 mm diameter samples. The discs were immersed in 100% ethanol for 2 s, placed in wells (24-well plate) and secured with a 15 mm O-ring (Viton 51414, Eriks) to prevent floating. This was followed by hydration steps in decreasing ethanol concentrations (90, 70, 60, 50, 40, 30, 15 and 10% ethanol) and finally 100% water, being the contact time for each concentration 2 s. The scaffolds were then sterilized by exposure to UV light for 30 min. Then, samples were incubated in 1 mL of DMEM without FBS for 3 h. Finally, the medium was removed, and the scaffolds were ready for cell seeding.

Human mesenchymal stem cells (hBM-MSCs) (Lonza Donor 38157, passage 5) were cultured in DMEM supplemented with 10% FBS, 1% penicillin-streptomycin and 5 mM of L-glutamine. Cells were washed with PBS and then tripsinized with 4 mL of trypsin/EDTA 0.25%. Cells were then centrifuged at 600× *g* for 5 min. The resulting pellet was suspended in 100 µL of culture medium and added drop by drop onto each scaffold (1 × 10^5^ cells/scaffold). Thereafter, 0.9 mL of DMEM were added per well, and scaffolds were incubated at 37 °C in 5% CO_2_ for different times. As a control, cells were cultured in TCP at the same density. Assays were also carried out with the pre-osteoblastic cell line MC3T3-E1 (subclone 4, CRL-2593; ETCC, Mannassas, VA, USA). In this case, cells were cultured in supplemented α-MEM, the number of cells for the scaffold assays was 4×10^4^ cells/scaffold and 2 × 10^4^ cells/well (24-well plate) for the Fe_3_O_4_-DMSA nanoparticles viability assay.

#### 2.8.1. Cell Viability Assay

hBM-MSCs and pre-osteoblastic cell growth onto the scaffolds was determined by fluorescence intensity with a Synergy4 Multimode Plate Reader (BioTek Instruments, Winooski, VT, USA) at 2 and 5 days with excitation and emission wavelengths of 560 and 590 nm, respectively. The Alamar Blue method (AbD Serotec, Oxford, UK) was used according to the manufacturer’s instructions. Scaffolds were exposed to 10% AlamarBlue^TM^ HS Cell Viability Reagent solution for 4 h at 37 °C in darkness before fluorescence measurements.

#### 2.8.2. Cell Adhesion and Spreading Assays

hBM-MSCs and pre-osteoblastic cell attachment, morphology and colonization onto the scaffolds were studied by SEM and fluorescence microscopy after 5 days of culture.

For the SEM study, the attached cells were rinsed twice with PBS and fixed with 4% (*w*/*v*) paraformaldehyde in PBS with 1% (*w*/*v*) sucrose at room temperature for 30 min. Thereafter, scaffolds were washed 3 times with PBS, dehydration was performed by slow water replacement by a series of graded ethanol solutions (30%, 50%, 70%, 80%, 90%, 96% ethanol), with final dehydration in absolute ethanol (100% ethanol, 2×). For the drying process, the dehydrated samples were immersed in hexamethyldisilazane (15 min, 2×) and dried overnight. The scaffolds were gold plated in a vacuum using a sputter coater.

For confocal microscopy, the cells adherent onto scaffolds were rinsed twice with PBS and fixed with 4% (*w*/*v*) paraformaldehyde in PBS with 1% (*w*/*v*) sucrose at 37 °C for 20 min, washed again with PBS and permeabilized with buffered 0.5% Triton X-100 (10.3 g sucrose, 0.292 g NaCl, 0.06 g MgCl_2_, 0.476 g HEPES, 0.5 mL of Triton X-100 in 100 mL of water, pH 7.2) at 4 °C for 5 min. Then, nonspecific bindings were blocked with 1% (*w*/*v*) bovine serum albumin (BSA) in PBS for 20 min at 37 °C. Samples were then incubated at 37 °C for 20 min with Atto 565-conjugated phalloidin (dilution 1:40), which stains actin filaments. After washing with PBS, cell nuclei were stained with DAPI in PBS. Confocal microscopy was analyzed with a confocal laser microscope OLYMPUS FV1200 (OLYMPUS, Tokyo, Japan), using a 60X FLUOR water dipping lens (NA = 1.0). Images were obtained using the software 3D Imaris to project a single 2D image–converted into a TIF file from the multiple Z sections by using an algorithm displaying the maximum pixel value of each Z 1-µm slice. DAPI and Atto 565-phalloidin staining were shown in blue and red, respectively.

## 3. Results and Discussion

### 3.1. Preparation and Characterisation of SPIONs

**Figure 1:**
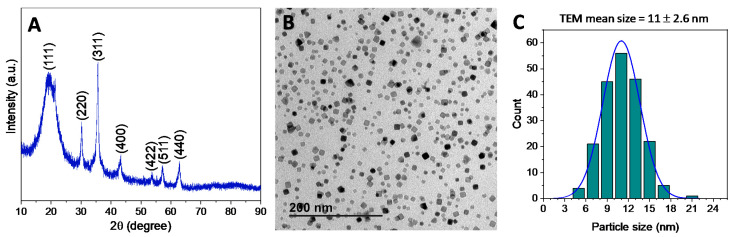
Powder X-ray diffraction pattern (**A**), TEM image (**B**) and NPs size distribution were obtained by statistical treatment of different TEM images (**C**) of Fe_3_O_4_-OA nanoparticles.

Iron oxide nanoparticles were prepared to exploit the thermal decomposition of iron (III) oleate as an iron-containing precursor [43,45]. After preparation, a comprehensive structural characterization of the oleic acid stabilized nanoparticles Fe_3_O_4_-OA was performed and the results are summarized in Figure 1.

In details, the XRD pattern of the resulting powders (Figure 1A) displays the characteristic maxima (2 2 0), (3 1 1), (4 0 0), (4 2 2), (5 1 1), and (4 4 0) of a Fe_3_O_4_ magnetite phase [46]. In addition, TEM analysis (Figure 1B) confirmed a fairly uniform nanoparticle size, with dimensions of ca. 11 nm, and octahedral or truncated octahedral nanoparticle morphologies. As further confirmation, Figure 1C shows the histogram of nanoparticle diameters (*n* = 200) as measured in TEM images with the best-fit lognormal distribution superimposed. The mean size and the standard deviation from the statistical analysis resulted in being 11.0 ± 2.6 nm, in agreement with the previous data. The normality test using the Kolmogorov-Smirnov approximation indicates that the data are significantly drawn from a normally distributed population. The dispersity value, or polydispersity index, of 0.24 was calculated as the ratio between the standard deviation and the mean of the particle diameter distribution, confirming that the size distribution of Fe_3_O_4_-OA nanoparticles presents a homogeneous population, as indicated by values lower than 0.3 [47].

The nanoparticles were further processed exploiting a ligand exchange of the oleic acid surface layer with DMSA to obtain a stable suspension of Fe_3_O_4_-DMSA nanoparticles in water [43]. Due to the hydrophobic nature of Fe_3_O_4_-OA nanoparticles, the ligand exchange is considered a necessary step for biological purposes. As confirmation of the successful functionalization, TGA performed on the Fe_3_O_4_-DMSA nanoparticles showed a weight loss of 15.3% in the 150 to 600 °C temperature range. Moreover, FTIR spectrum (Figure S1) presented a weak absorption peak at 2916 cm^−1^ (due to the aliphatic ν_(CH)_ modes) and broad bands at 1574 and 1365 cm^−1^ related to the asymmetric and symmetric stretching of the –COO^−^ groups of DMSA respectively, confirming the successful ligand interchange. 

As further confirmation, the DLS measurement of the resulting aqueous suspension indicated a monomodal hydrodynamic size distribution centered at 21 nm (Figure S2). The ζ-potential value of −26.7 mV (measured at pH 7) also indicated that most carboxylic groups at the surface of the nanoparticles are deprotonated, suggesting that the long-term colloidal stability in water can be ascribed to the electrostatic repulsion among –COO^−^ groups.

### 3.2. Design and Characterisation of N_COL/SPIONs Electrospun Scaffolds

**Figure 2:**
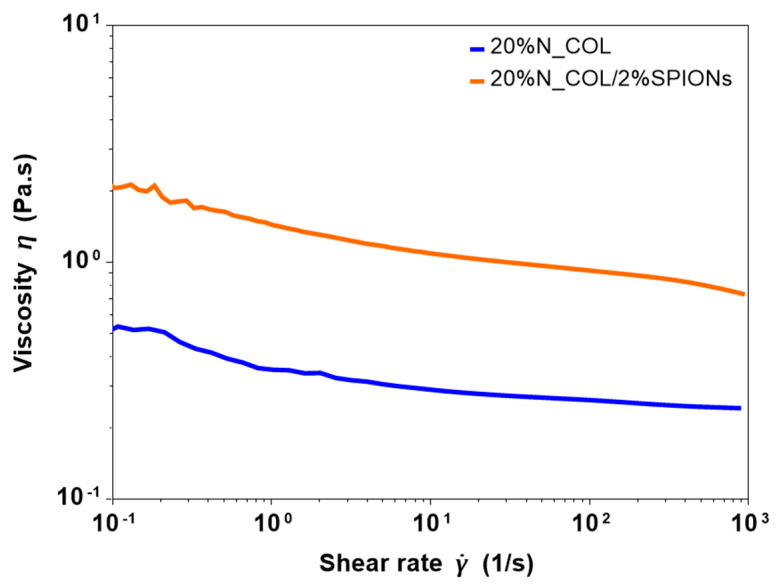
Viscosity of collagen formulation before (20% N_COL) and after (20% N_COL/2% SPIONs) the incorporation of the SPIONs.

The possibility to use superparamagnetic nanoparticles to create hybrid formulations suitable for the electrospinning of biocompatible 3D smart collagen-based scaffolds has been explored. To this purpose, before performing the electrospinning tests, rheological analyses on the collagen formulation before and after the addition of SPIONs were conducted to measure the viscosity and the overall processability of the systems. As presented in Figure 2, flow ramp tests were conducted, observing the variation of the viscosity at increasing shear rates (10^−^^1^–10^3^ s^−^^1^) at a constant temperature of 23 °C, to mimic the environmental conditions used during the electrospinning process. As proved by the curves reported in Figure 2, the introduction of SPIONs did not significantly alter the rheological properties of the system, where both 20% N_COL and 20% N_COL/2% SPIONs showed a similar shear thinning behaviour, described by the decrease of viscosity at higher shear rates. A slight increase of the viscosity from about 0.5 Pa.s to 2 Pa.s was registered after the addition of SPIONs. Despite the high concentration of collagen, the analysis registered low values of viscosity of the systems, confirming the high solubility of the protein in the selected solvent.

**Figure 3:**
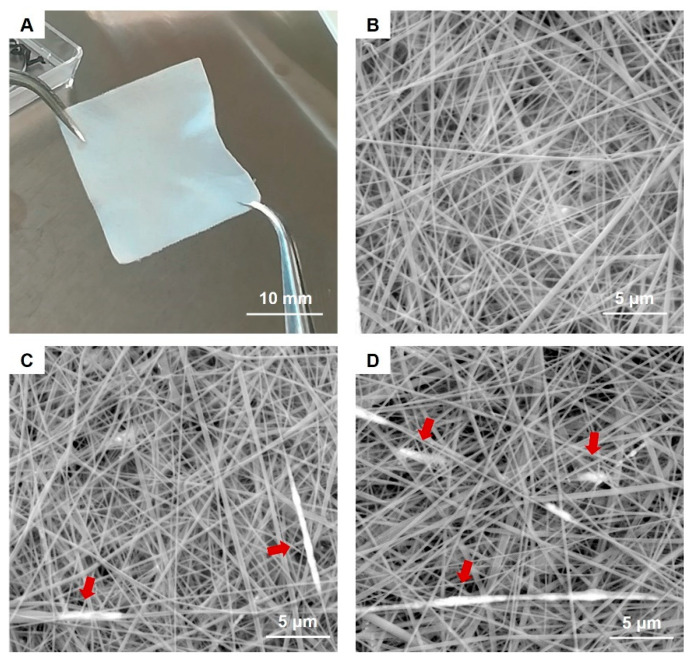
Image of the resulting 20 × 20 mm 20% N_COL/2% SPIONs scaffold (**A**) and SEM images of 20% N_COL (**B**) and 20% N_COL/2% SPIONs (**C**,**D**) showing the fibrous structure at the microscale. Red arrows indicate the presence of SPIONs along the fibers.

In addition, exploratory tests performed at first with the SPIONs-free collagen formulation evidenced the good processability of the selected collagen solution (20% N_COL) by means of electrospinning, leading to homogeneous nanostructured matts characterised by fibres between 100–200 nm in diameter (see Figure 3B).

Based on these promising results, SPIONs were incorporated into the collagen solution, resulting in a final weight percentage of 2% with respect to the collagen weight (20% N_COL/2% SPIONs). The concentration of SPIONs was selected according to the range already reported in the literature [33], with the purpose of achieving magnetically responsive scaffolds while preserving the biocompatibility of the final systems.

The developed 20% N_COL/2% SPIONs formulation was subsequently processed by means of electrospinning, by using a plate collector to obtain random-like fibrous mats.

For the scaffold preparation, the jet was stabilised, exploiting an applied voltage and material flow of 20 kV and 300 μL/h respectively, keeping constant conditions of temperature and humidity for 3 h. Similarly, the 20% N_COL formulation was processed exploiting applied voltage and flow rate of 22 kV and 400 μL/h respectively, where the resulting scaffolds were used as reference for the further characterisation in terms of morphological, magnetic and biological properties.

To increase the stability of the constructs and avoid a too fast dissolution in aqueous medium, the obtained 20% N_COL and 20% N_COL/2% SPIONs mats were subsequently chemically crosslinked upon incubation in a solution of 30 mM EDC and 15 mM NHS in ethanol, following a procedure previously reported in the literature [44].

The morphological assessment of the resulting scaffolds (Figure 3A) was subsequently investigated by means of SEM.

**Figure 4:**
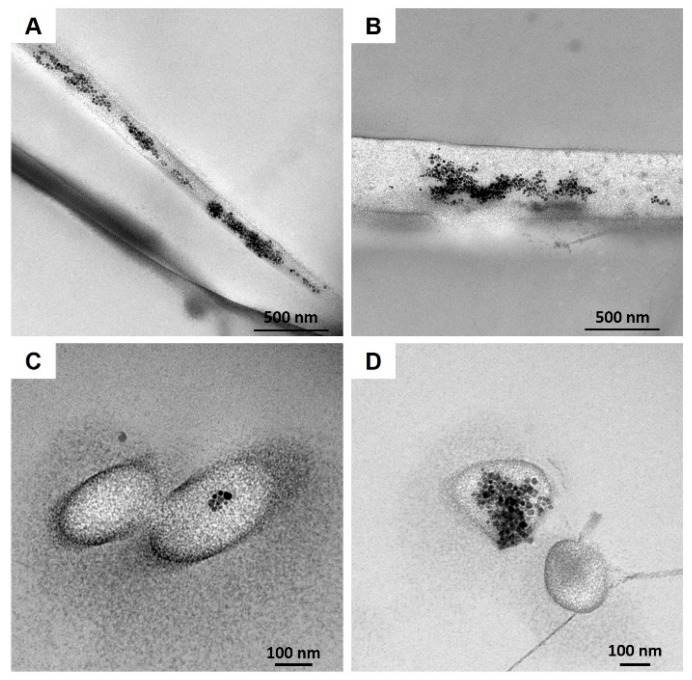
TEM images of 20% N_COL/2% SPIONs highlighting the SPION distribution into the collagen fibers: horizontal (**A**,**B**) and vertical sections (**C**,**D**).

SEM images clearly showed the nanostructure of the 20% N_COL/2% SPIONs scaffolds (Figure 3C,D), characterized by randomly oriented fibres of about 170 nm, evidencing a slight increase in the final fibre diameter compared to the electrospinning of collagen alone (Figure 3B). SEM analysis confirmed the formation of a homogeneous biomimetic system presenting fibres with homogeneous dimension and morphology, mimicking the microarchitectural features of the native ECM. Small aggregates of SPIONs were visible throughout the scaffolds, along the collagen fibers (Figure 3C,D). However, due to the extremely small size of the particles, TEM was exploited to better explore the distribution and the aggregate dimension of SPIONs (Figure 4). As shown in Figure 4, TEM images of both horizontal and vertical sections of the 20% N_COL/2% SPIONs scaffolds confirmed the embedding of the superparamagnetic particles into the collagen fibers with aggregates not larger than 200 nm in size, without non-significant distortion of both fiber morphology and dimension. The natural high tendency of nanoparticles to aggregate due to their large surface tension and the electrostatic forces involved in the electrospinning process is expected to favour the formation of aggregates. However, the good stability of the Fe_3_O_4_-DMSA in the solvent and their further dispersion into the collagenous system avoided the massive aggregation of nanoparticles during the process, as confirmed by the obtained data.

**Figure 5:**
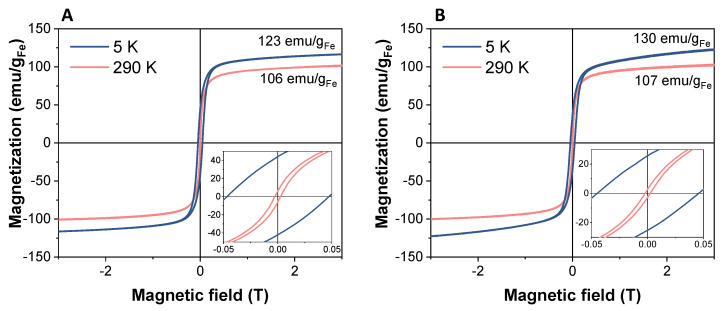
Magnetization curves of Fe_3_O_4_-DMSA nanoparticles (**A**) and 20% N_COL/2% SPIONs scaffold (**B**) normalized to the grams of iron. The magnification of the curves at low fields is shown in the insets.

With regard to the magnetic properties of the materials, SPIONs exhibited magnetization curves with similar shape and coercivity at low and room temperatures, both alone or incorporated into the collagen scaffold (Figure 5A,B). As shown in the graphs, coercivity values lower than 3 mT were registered in both cases at room temperature, which for most of the biomedical applications of the SPIONs can be considered as a non-hysteric response, confirming their superparamagnetic behaviour (see insets in Figure 5). At low temperature, the coercivity is 450 Oe for both samples, thus registering no significant differences between nanoparticles alone and SPION-containing scaffolds. Therefore, the similarity between both cycles suggests that the magnetic properties of SPIONs were not affected by their integration into collagen scaffolds. 

Furthermore, the Fe_3_O_4_-OA sample showed a magnetization curve very similar to that presented by the DMSA-coated nanoparticles, indicating that the magnetic properties are also unaffected after ligand exchange (see Figure S3B and Figure 5A).

Finally, the maximum magnetization values normalized to Fe content calculated for SPIONs alone and Coll/SPIONs scaffolds resulted to be consistent, M_SPIONs_ (3 T, RT-5K) = 106–123 emu/gFe and M_SPIONs-Scaffold_ (3 T, RT-5K) = 107–130 emu/gFe, confirming that the integration into the collagen matrix does not affect the magnetic response of the nanoparticles. The magnetization value at 3 T normalized to the total mass of scaffold is reduced to 0.53 emu/g (Figure S3A), as expected, considering that collagen represents 99.4% of the overall weight (based on the amount of Fe as measured by ICP).

### 3.3. Biological Assessment

**Figure 6:**
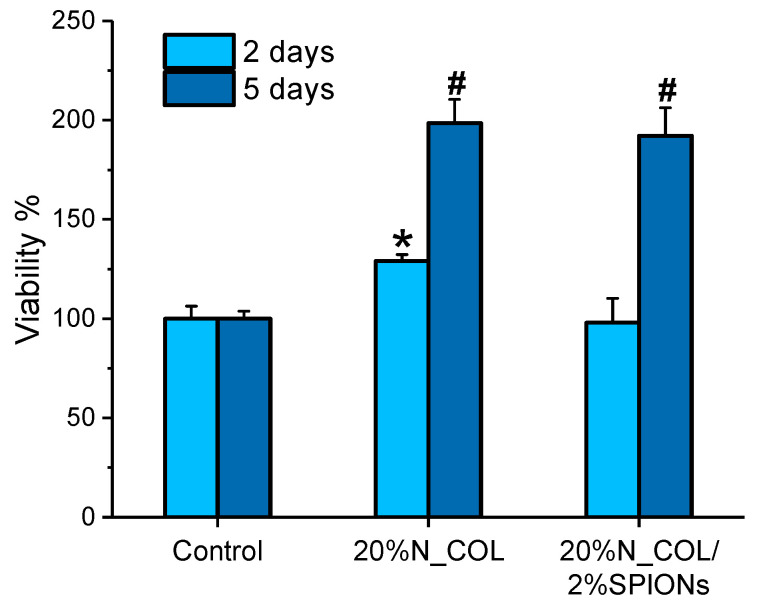
Cell viability of hMSCs incubated onto 20% N_COL and 20% N_COL/2% SPIONs scaffolds after 2 and 5 days (measured by Alamar Blue). * indicate *p* < 0.01 vs. control; # indicate *p* < 0.001 vs. control.

With the aim of demonstrating whether the incorporation of magnetic nanoparticles into electrospun collagen scaffolds influences cell viability, spreading and colonization of the scaffolds, a preliminary cytocompatibility assay was performed using two types of cells, namely hMSCs and MC3T3-E1. Figure 6 shows the proliferation of hMSCs cultured onto 20% N_COL and 20% N_COL/2% SPIONs scaffolds at 2 and 5 days, measured by the Alamar Blue method. On day 2, a slight increase in the percentage of cell viability was observed for electrospun collagen scaffolds (20% N_COL) compared to the control (TCP-cultured cells). This observation could be attributed to the nanostructure of the matrix as well as the ability of collagen to enhance cell activity and promote cell adhesion thanks to specific aminoacidic sequences such as RGD and the hexapeptide GFOGER [36]. Similarly, SPIONs containing scaffolds overall preserved cell viability with respect to the control. On day 5, hMSCs cultured onto scaffolds without and with SPIONs equally exhibited notable increase in proliferation, demonstrating that the addition of SPIONs into the collagenous matrix does not affect the final biocompatibility of the system. Furthermore, these results were found to be consistent to those obtained with MC3T3-E1 cells (Figure S4), which indicated good preservation of cell viability for SPION-containing scaffolds. These findings are also coherent with those obtained for nanoparticles alone at different concentrations (Figure S5).

**Figure 7:**
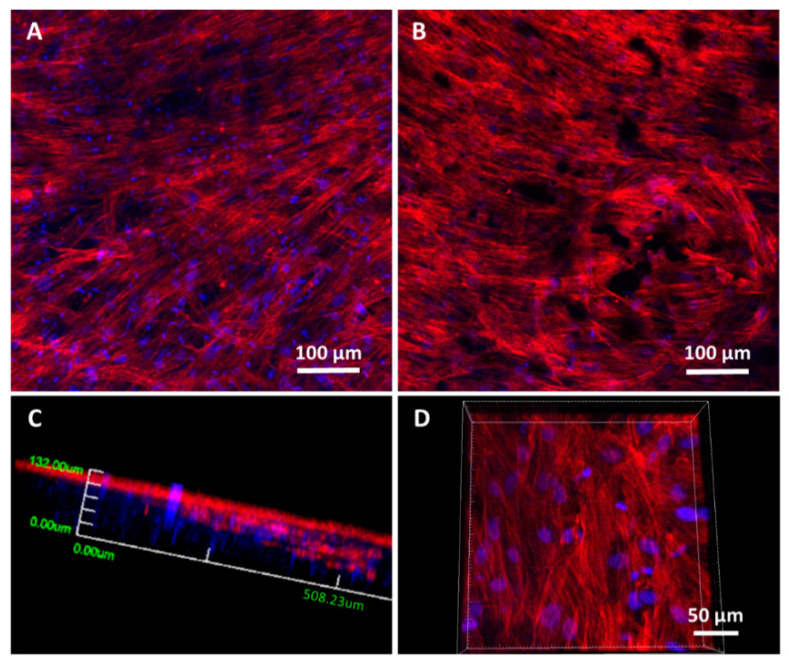
Representative confocal laser scanning microscopy images of hMSCs cultured onto 20% N_COL (**A**) and 20% N_COL/2% SPIONs (**B**–**D**) scaffolds for 5 days. F-actin microfilaments were stained with Atto 565-phaloidin to visualize the cytoskeleton and determine cell morphology (red fluorescence). Nuclei were stained with DAPI (blue fluorescence). Images were generated using Z-axis projections (**A**,**B**). (**C,D**) 3D-reconstruction and different magnification of the z-projection corresponding to 20% N_COL/2% SPIONs electrospun scaffold. (**C**) 3D reconstruction at low magnification taken from the surface until a z-height of 200 microns. (**D**) 3D reconstruction at higher magnification taken from the center of the scaffold thickness down to 100 microns.

Spreading, colonization and cell morphological studies were carried out on 20% N_COL and 20% N_COL/2% SPIONs scaffolds by confocal laser scanning microscopy (Figure 7). Different parts of the scaffolds were scanned after 5 days of culture, using Atto 565-phalloidin as a fluorescence probe for F-actin microfilaments and DAPI fluorostaining for nuclei. The images showed that hMSCs presented their typical spindle-shaped morphology, spread over the surface of the 20% N_COL and 20% N_COL/2% SPIONs scaffolds and colonized the entire scaffold without leaving any gaps (Figure 7A,B, respectively) in agreement with what was observed for MC3T3-E1 cells (Figure S6). Moreover, confocal microscopy enabled observation of the presence of cells throughout the entire thickness of the scaffold by taking images at different depths (z-heights). A three-dimensional reconstruction of the different scaffolds was carried out, showing complete colonization of the constructs. Figure 7C shows a representative image at low magnification of the SPION-containing scaffolds. As a detail, a higher magnification image is also shown in Figure 7D, where the cellular lattice arranged throughout the thickness of the scaffold can be clearly observed (see Video S1 in the Supplementary Materials). The results showed complete colonization forming an adequate and complete cellular lattice regardless of the presence of embedded SPIONs.

**Figure 8:**
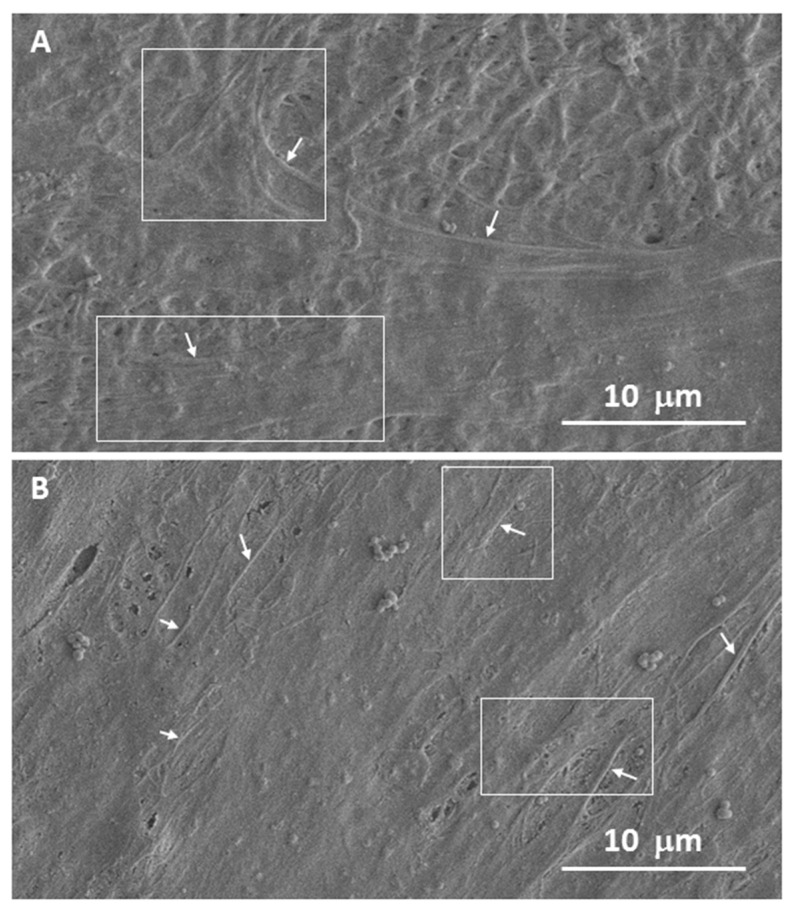
Representative SEM micrographs of hMSCs cultured onto 20% N_COL (**A**) and 20% N_COL/2% SPIONs (**B**) after 5 days of incubation. The areas highlighted in the images with arrows and boxes indicate the different projections of these cells in the form of filopodia and lamellipodia, respectively.

Cell adhesion, spreading and morphology after 5 days of incubation of both electrospun scaffolds have also been evaluated by SEM (see Figure 8 and Figure S7). After 5 days of culture, the cells appeared to be fully integrated into the scaffold structure, extending their typical projections (filopodia and lamellipodia) into and around the nanofibers (Figure 8A) also in the presence of dispersed SPIONs (Figure 8B). This interaction demonstrates that the cells are not only able to adhere to the surface but are also able to proliferate within the electrospun scaffolds, as demonstrated by confocal analysis.

The results shown in Figure 7 and Figure 8 show that after 5 days, the results obtained in the viability test (Figure 5) are corroborated where both electrospun matrices are similarly colonized on their whole surface, demonstrating that the presence of SPIONs does not alter the overall cellular response.

## 4. Conclusions

In this work, a nanostructured magnetic scaffold composed of type-I collagen embedding superparamagnetic iron oxide nanoparticles (SPIONs) has been successfully obtained by electrospinning. To this aim, SPIONs prepared by thermal decomposition and ligand exchange have been homogeneously incorporated into a type-I collagen solution thanks to their enhanced colloidal stability in water medium.

The rheological properties of the system were not significantly altered by the introduction of SPIONs with respect to the SPIONs-free collagen solution investigated as a reference in view of the electrospinning processing. The hybrid formulation has been successfully processed by electrospinning, resulting in nanostructured fibrous mats. The morphological characterization of the scaffolds by means of SEM and TEM confirmed the successful incorporation of the SPIONs, which resulted in being properly embedded in the collagen fibers.

In addition, the magnetic characterization indicated that the superparamagnetic properties of the SPIONs are preserved after their incorporation into the collagen scaffold.

The selected amount of incorporated SPIONs (2% *w*/*w* with respect to the collagen weight) was effective in obtaining a saturation magnetization value of 0.53 emu/g referred to the total scaffold weight.

Moreover, the biological assessment performed on the scaffolds proved high viability, adhesion, and proliferation of both hMSCs and MC3T3-E1 cells, proving the high biocompatibility of the final system for the envisaged biomedical applications.

The architecture obtained by electrospinning in combination with the superparamagnetic behaviour of the incorporated SPIONs make the developed electrospun scaffolds very promising tools for advanced and personalized treatments for bone regeneration, and for this ultimate goal, future in-depth studies applying different magnetic fields and their influence on cell differentiation behaviour are underway.

## Data Availability

The data presented in this study are openly available in ZENODO at 10.5281/zenodo.5812097, https://doi.org/10.5281/zenodo.5812097.

## Supplementary Materials

The following supporting information can be downloaded at https://www.mdpi.com/article/10.3390/nano12020181/s1, Figure S1: FTIR spectrum of Fe_3_O_4_-DMSA nanoparticles; Figure S2: Hydrodynamic size distribution obtained by dynamic light scattering of Fe_3_O_4_-DMSA nanoparticles suspended in water; Figure S3: Magnetization curve of Fe_3_O_4_-OA nanoparticles with magnetic saturation value in emu/g of iron; Figure S4: Cell viability of MC3T3-E1 onto 20% N_COL and 20% N_COL/2% SPIONs scaffolds measured by Alamar Blue at 2 and 5 days; Figure S5: Viability of MC3T3-E1 preosteoblast-like cells in contact with different concentrations of Fe_3_O_4_-DMSA nanoparticles for 2 h and measured by Alamar Blue at 1 and 4 days of cell culture; Figure S6: Representative confocal laser images of MC3T3-E1 cells cultured onto 20% N_COL and 20% N_COL/2% SPIONs scaffolds for 5 days; Figure S7: Confocal and SEM images of hMSCs onto 20% N_COL and 20% N_COL/2% SPIONs scaffolds for 5 days; Video S1: 3D reconstruction by confocal microscope of 20% N_COL/2% SPIONs scaffolds incubated with hMSC during 5 days, the reconstruction have performed compiling different heights in Z.
