# Supplementary material for: Incorporation of Superparamagnetic Iron Oxide Nanoparticles into Collagen Formulation for 3D Electrospun Scaffolds
Source: Nanomaterials (Basel). 2022 Jan 6;12(2):181. doi: 10.3390/nano12020181 (PMC8778221; doi:10.3390/nano12020181)
Supplement: Supplementary file 1 [file nanomaterials-12-00181-s001.zip › nanomaterials-1469208-supplementary.pdf]

Supplementary Materials

Incorporation of Superparamagnetic Iron Oxide Nanoparticles into Collagen Formulation for 3D Electrospun Scaffolds

Manuel Estévez [1,†], Giorgia Montalbano [2,†], Alvaro Gallo-Cordova [3], Jesús G. Ovejero [3], Isabel Izquierdo-Barba [1,4,*], Blanca González [1,4], Clarissa Tomasina [5], Lorenzo Moroni [5], María Vallet-Regí [1,4], Chiara Vitale-Brovarone [2] and Sonia Fiorilli [2,*]

[1] Departamento de Química en Ciencias Farmacéuticas, Facultad de Farmacia, Universidad Complutense de Madrid, 28040 Madrid, Spain; Instituto de Investigación Sanitaria Hospital 12 de Octubre i+12; manestev@ucm.es (M.E.); blancaortiz@ucm.es (B.G.); vallet@ucm.es (M.V.-R.)
[2] Department of Applied Science and Technology, Politecnico di Torino, 10129 Torino, Italy; giorgia.montalbano@polito.it (G.M.); chiara.vitalebrovarone@polito.it (C.V.-B.)
[3] Department of Energy Environment and Health, Instituto de Ciencia de Materiales de Madrid C.S.I.C. Sor Juana Inés de la Cruz 3, Cantoblanco, 28049 Madrid, Spain; alvaro.gallo@csic.es (A.G.-C.); jgovejero@ucm.es (J.G.O.)
[4] CIBER de Bioingeniería Biomateriales y Nanomedicina CIBER-BBN, 28040 Madrid, Spain
[5] Complex Tissue Regeneration Department, MERLN Institute for Technology-Inspired Regenerative Medicine, Maastricht University, Universiteitssingel 40, 6229 ET Maastricht, The Netherlands; c.tomasina@maastrichtuniversity.nl (C.T.); l.moroni@maastrichtuniversity.nl (L.M.)
* Correspondence: ibarba@farm.ucm.es (I.I.-B.); sonia.fiorilli@polito.it (S.F.)
† These authors contributed equally to this work.

Supplementary Materials:

Figure S1: FTIR spectrum of Fe_3O_4-DMSA nanoparticles; **Figure S2:** Hydrodynamic size distribution obtained by dynamic light scattering of Fe_3O_4-DMSA nanoparticles suspended in water; **Figure S3:** Magnetization curve of Fe_3O_4-OA nanoparticles at 290 K (A) and magnetization curve of 20%N_COL/2%SPION scaffold at 290 K and 5 K with magnetic saturation value in emu/g of scaffold; **Figure S4:** Cell viability of MC3T3-E1 onto 20%N_COL and 20%N_COL/2%SPIONs scaffolds measured by Alamar Blue at 2 and 5 days; **Figure S5:** Viability of MC3T3-E1 preosteoblast-like cells in contact with different concentrations of Fe_3O_4-DMSA nanoparticles for 2 hours and measured by Alamar Blue at 1 and 4 days of cell culture; **Figure S6:** Representative confocal laser scanning microscopy images of MC3T3-E1 cells cultured onto 20%N_COL and 20%N_COL/2%SPIONs scaffolds for 5 days. **Figure S7:** Confocal and SEM images of hMSCs onto 20%N_COL and 20%N_COL/2%SPIONs scaffolds for 5 days; **Video S1:** 3D reconstruction by confocal microscopy of 20%N_COL/2%SPIONs scaffolds incubated with hMSC during 5 days, the reconstruction have performed compiling different heights in Z.

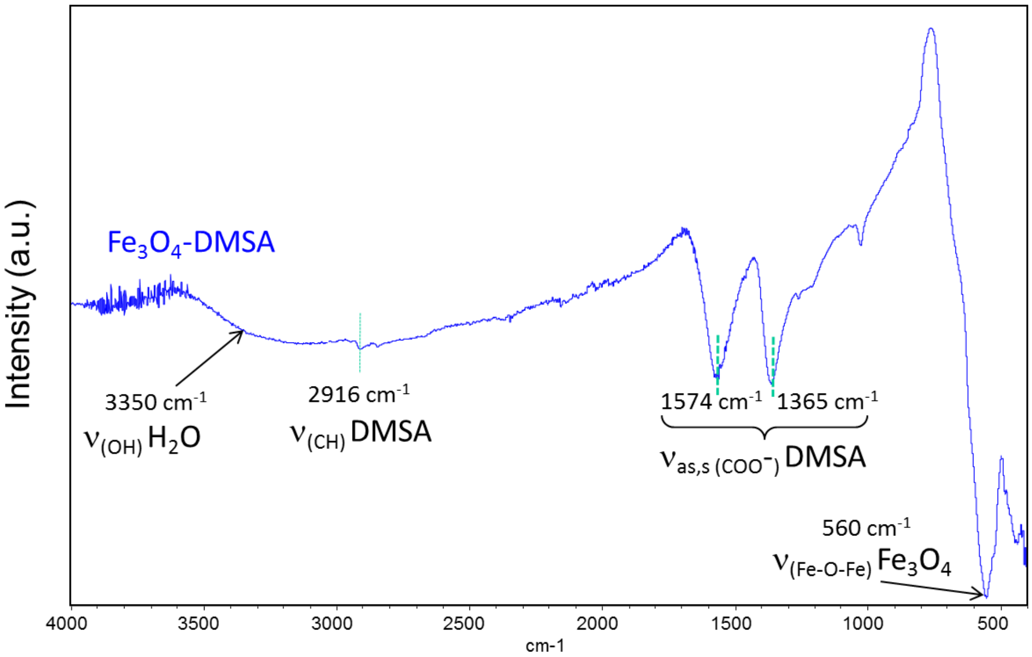

Figure S1. FTIR spectrum of Fe_3O_4-DMSA nanoparticles in the 4000–400 cm^{-1} region.

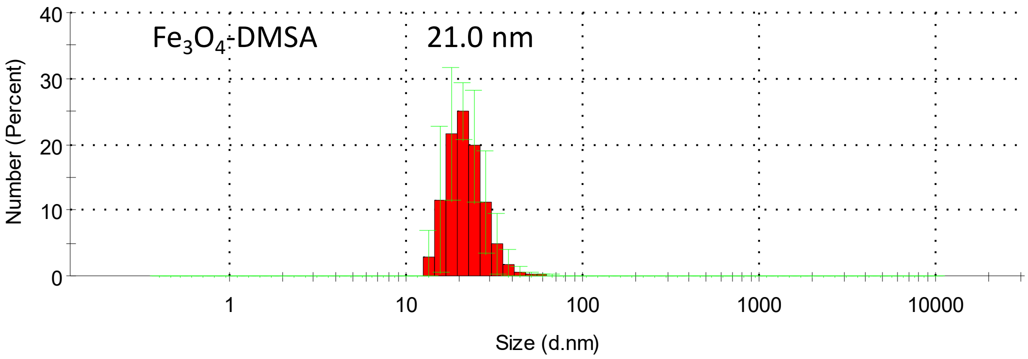

Figure S2. Hydrodynamic size distribution obtained by dynamic light scattering of Fe_3O_4-DMSA nanoparticles suspended in water.

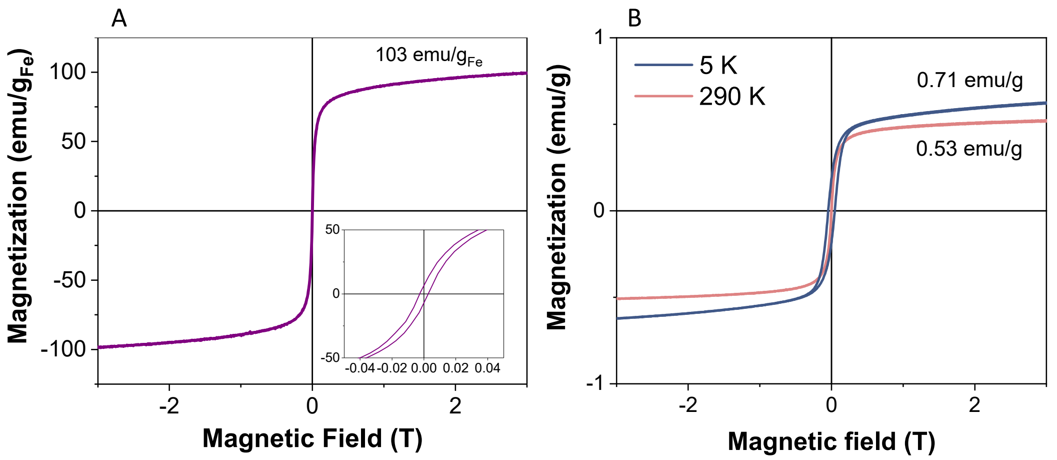

Figure S3. Magnetization curve of Fe_3O_4-OA nanoparticles at 290 K with magnetic saturation value in emu/g of iron (**A**) and magnetization curve of 20%N_COL/2%SPION scaffold at 290 K and 5 K with magnetic saturation value in emu/g of scaffold (**B**). The magnification of the magnetization curve of Fe_3O_4-OA nanoparticles is shown in the inset.

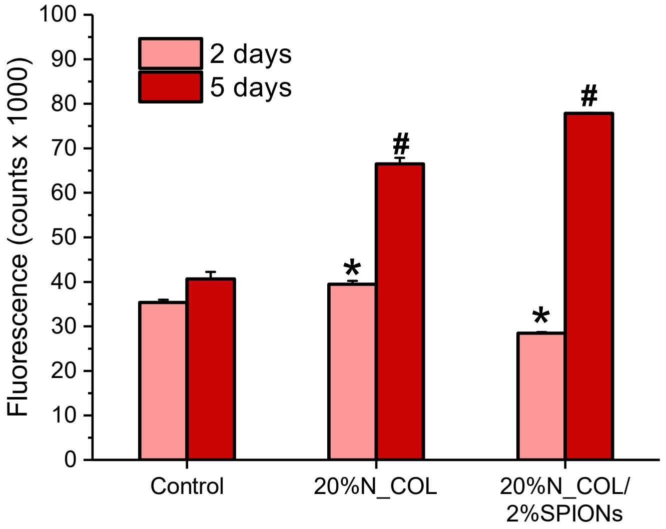

Figure S4. P Cell viability of MC3T3-E1 onto 20%N_COL and 20%N_COL/2%SPIONs scaffolds measured by Alamar Blue at 2 and 5 days. * indicate p < 0.05 vs control; # indicate p < 0.01 vs control.

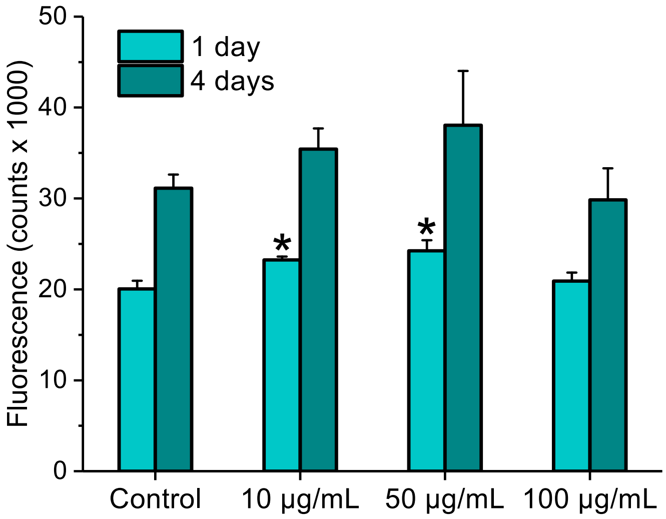

Figure S5. Viability of MC3T3-E1 preosteoblast-like cells in contact with different concentrations of Fe_3O_4-DMSA nanoparticles for 2 hours and measured by Alamar Blue at 1 and 4 days of cell culture. * indicate $p < 0.05$ vs control.

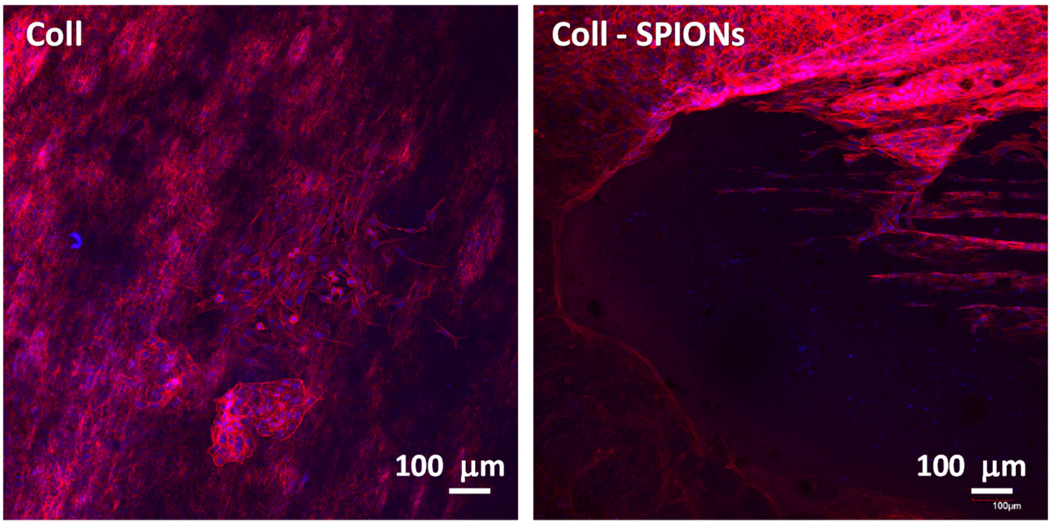

Figure S6. Representative confocal laser scanning microscopy images of MC3T3-E1 cells cultured onto 20%N_COL and 20%N_COL/2%SPIONs scaffolds for 5 days. F-actin microfilaments were stained with Atto 565-phaloidin to visualize the cytoskeleton and determine cell morphology (red fluorescence). Nuclei were stained with DAPI (blue fluorescence).

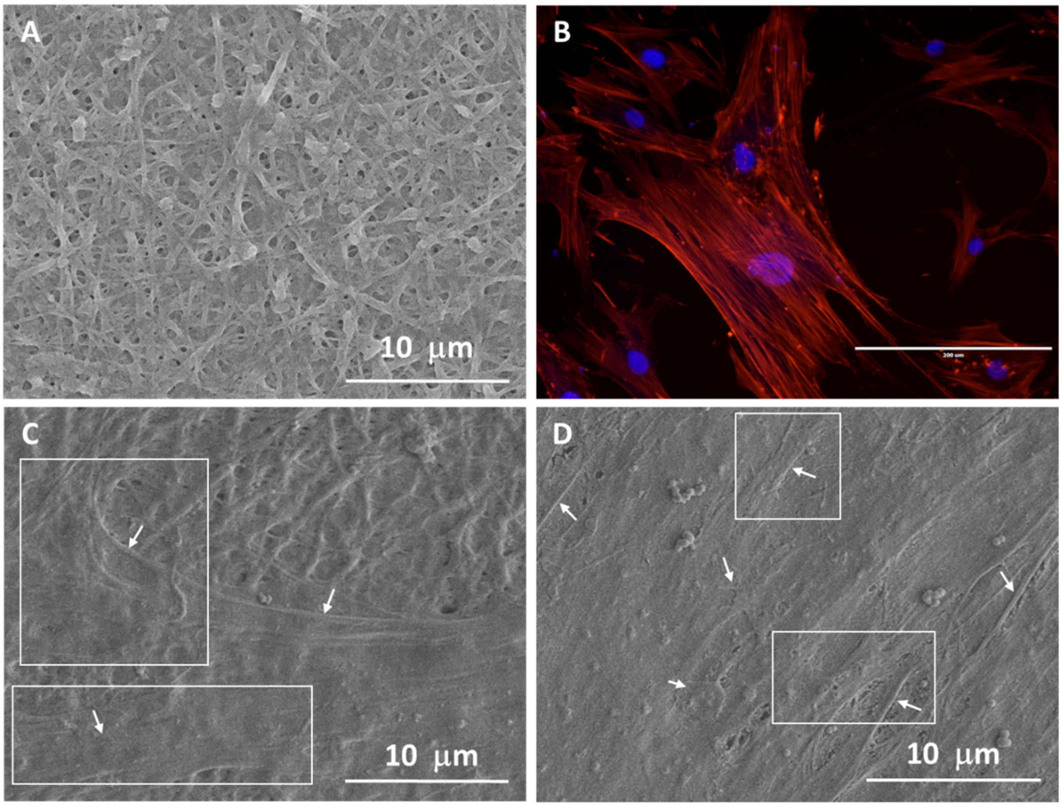

Figure S7. Image **A** is a representative SEM micrograph of the 20%N_COL/2%SPIONs material after 5 days of incubation in culture medium, without cells. **B** is a fluorescence microscopy image of hMSCs cultured in TCP for 5 days where F-actin microfilaments were stained with Atto 565-phalloidin to visualise the cytoskeleton and determine cell morphology (red fluorescence), nuclei were stained with DAPI (blue fluorescence); image was taken using an EVOS FL inverted fluorescence microscope set at 20× magnification. **C** and **D** are SEM micrographs of hMSCs cultured onto 20%N_COL and 20%N_COL/2%SPIONs respectively, after 5 days of incubation. The areas highlighted in the images with arrows and boxes indicate the different projections of these cells in the form of filopodia and lamellipodia respectively. The image panel clearly shows that the cell morphology visible in the confocal image corresponds to that observed in the SEM images.
